# Supplementary material for: Expressing the Pro-Apoptotic Reaper Protein via Insertion into the Structural Open Reading Frame of Sindbis Virus Reduces the Ability to Infect Aedes aegypti Mosquitoes
Source: Viruses. 2022 Sep 13;14(9):2035. doi: 10.3390/v14092035 (PMC9501589; doi:10.3390/v14092035)
Supplement: Supplementary file 1 [file viruses-14-02035-s001.zip › Figure S1.pdf]

Figure S1

A. Carpenter and R.J. Clem, Expressing the pro-apoptotic Reaper protein via insertion into the structural open reading frame of Sindbis virus reduces the ability to infect *Aedes aegypti* mosquitoes

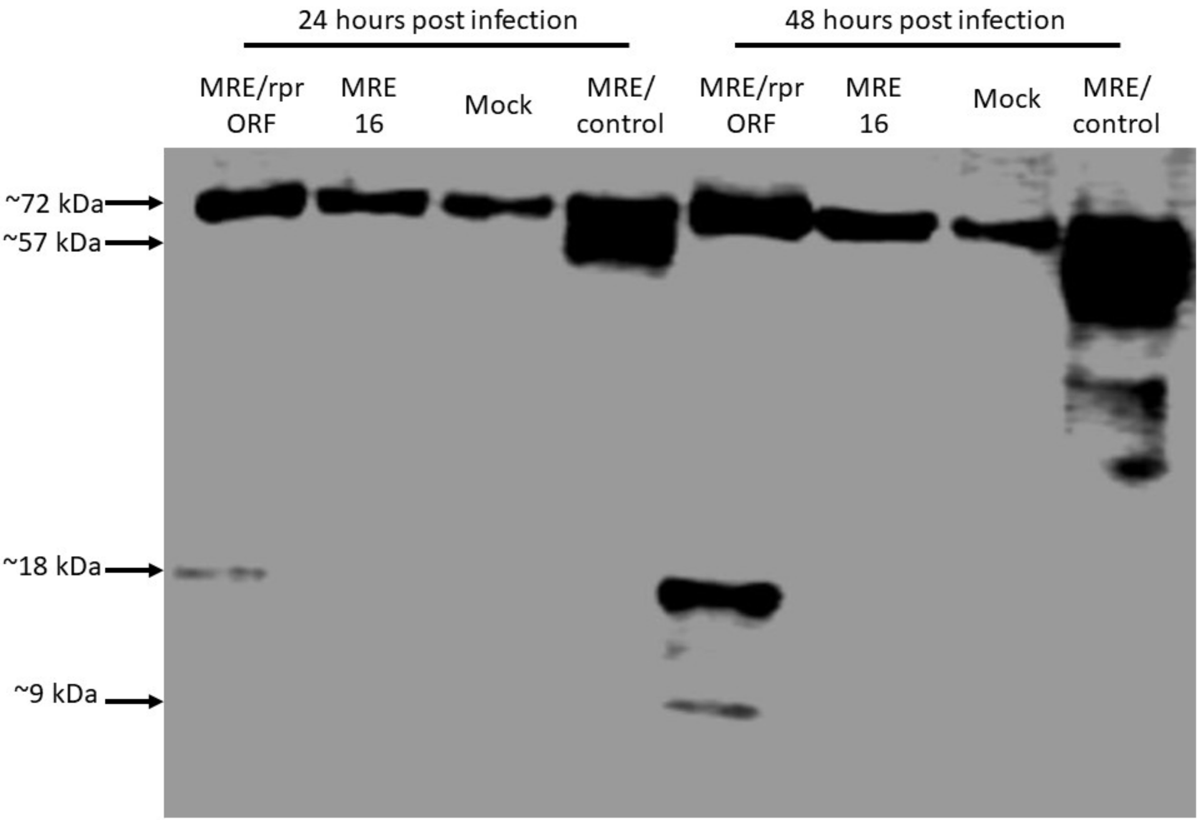

Figure S1. Image of full immunoblot from Fig. 2. C6/36 cells were infected with MRE/rprORF, 5’dsMRE16ic (labelled MRE16), MRE/control or were mock infected and protein was extracted at 24 and 48 hpi. Immunoblotting was done using anti-HA antibody.
